# Relating Stool Microbial Metabolite Levels, Inflammatory Markers and Dietary Behaviors to Screening Colonoscopy Findings in a Racially/Ethnically Diverse Patient Population

**DOI:** 10.3390/genes9030119

**Published:** 2018-02-26

**Authors:** Kristina M. Bridges, Francisco J. Diaz, Zhiwen Wang, Ishfaq Ahmed, Debra K. Sullivan, Shahid Umar, Daniel C. Buckles, K. Allen Greiner, Christina M. Hester

**Affiliations:** 1 Department of Family Medicine Research Division, University of Kansas Medical Center, Kansas City, KS 66160, USA; kbridges@kumc.edu (K.M.B.); agreiner@kumc.edu (K.A.G.); 2 Department of Biostatistics, University of Kansas Medical Center, Kansas City, KS 66160, USA; fdiaz@kumc.edu (F.J.D.); zwang3@kumc.edu (Z.W.); 3 Department of Surgery, University of Kansas Medical Center, Kansas City, KS 66160, USA; iahmed@kumc.edu (I.A.); sumar@kumc.edu (S.U.); 4 Department of Dietetics and Nutrition, University of Kansas Medical Center, Kansas City, KS 66160, USA; dsulliva@kumc.edu; 5 University of Kansas Cancer Center, Kansas City, KS 66160, USA; 6 Department of Internal Medicine, Gastroenterology and Hepatology, University of Kansas Medical Center, Kansas City, KS 66160, USA; dbuckles@kumc.edu

**Keywords:** colorectal cancer, short chain fatty acids, diet, inflammation, microbiota, health disparities, colonoscopy

## Abstract

Colorectal cancer (CRC) is the third leading cause of cancer death for both men and women in the United States, yet it is treatable and preventable. African Americans have higher incidence of CRC than other racial/ethnic groups, however, it is unclear whether this disparity is primarily due to environmental or biological factors. Short chain fatty acids (SCFAs) are metabolites produced by bacteria in the colon and are known to be inversely related to CRC progression. The aim of this study is to investigate how stool SCFA levels, markers of inflammation in stool and dietary intake relate to colonoscopy findings in a diverse patient population. Stool samples from forty-eight participants were analyzed for SCFA levels and inflammatory markers (lysozyme, secretory IgA, lactoferrin). Additionally, participants completed the National Cancer Institute’s Diet History Questionnaire II (DHQ II) to report dietary intake over the past year. Subsequently, the majority of participants underwent screening colonoscopy. Our results showed that African Americans had higher total levels of SCFAs in stool than other racial/ethnic groups, significantly lower intake of non-starchy vegetables and similar inflammatory marker expression and colonoscopy outcomes, compared to others. This work is an initial exploration into the biological and clinical factors that may ultimately inform personalized screening approaches and clinical decision-making to improve colorectal cancer disparities for African Americans.

## 1. Introduction

Colorectal cancer (CRC) is the third leading cause of cancer death overall in the United States [1]. CRC incidence and death rates vary by race/ethnicity; African Americans (AA) suffer the greatest incidence and mortality rate and are diagnosed at later stages than other racial/ethnic groups in the US, despite comparable screening rates [2,3]. Further, the magnitude of disparity for mortality is twice as high as that for incidence between non-Hispanic African Americans and non-Hispanic Whites [2]. Though environmental and biological factors, including diet, gut microbiota and intestinal inflammation have been linked to progression and prevention of CRC, it is unclear whether these factors play a role in CRC outcome disparities between AA and others. 

The gut microbiota, the more than 1 trillion bacteria inhabiting the intestinal tract, and the metabolites they produce, including short chain fatty acids (SCFAs), may be important modifiable factors related to CRC risk. Unfortunately, little is known about microbes and the pre-cancerous developmental processes of the normal human colon. We do know, however, that individuals diagnosed with CRC have modified bacterial profiles and different levels of bacterial metabolites such as SCFAs as compared to healthy controls [4,5,6,7,8,9,10,11,12,13]. In a small pilot study with average risk adults (5 AA, 5 Hispanic, 5 Native American and 5 White participants), we previously showed AAs to have significantly lower stool acetate, butyrate and total SCFA levels than other racial/ethnic groups and to have a significantly higher Firmicutes/Bacteroidetes ratio [14]. In contrast, one recent study found higher SCFAs in the stool of AAs with adenomatous polyps than in the stool of healthy AAs [15].

SCFAs are the preferred energy source of colonic epithelial cells and have been shown to directly influence cell signaling and promote or suppress tumorigenesis among colonic epithelial cells [16]. Butyrate is the most well-studied SCFA and has been shown to have anti-proliferative and anti-cancerous properties in in vitro studies and mouse models respectively [17]. While SCFAs seem to be protective, evidence supports both protective and harmful roles for bacteria and their metabolites in CRC [18]. The exact role of bacteria in CRC development is still unclear and is likely mediated in large part by diet [17,19].

Evidence suggests that dietary intake can promote CRC (red meat, processed meat) or prevent CRC (fiber, fruits and non-starchy vegetables) [19]. Recent studies have shown that diet modification may reduce CRC risk. For example, switching African Americans to a high fiber, low fat diet for two weeks was associated with an increase in SCFAs and a significant reduction in colonic mucosal inflammation and proliferation of biomarkers of cancer risk; whereas switching native Africans to a high fat, low fiber diet resulted in reciprocal changes [20].

While microbial influence on CRC is the subject of intense investigation, intestinal inflammation is known to promote conditions that favor the development of CRC [21,22]. Inflammatory bowel disease (IBD; i.e., Crohn’s disease, ulcerative colitis) increases CRC risk by as much as ten-fold [21]. Dysregulation of the immune system in the colon can lead to IBD or underlying, chronic inflammation [21]. Inflammation in the colon results in elevated levels of lactoferrin and lysozyme in stool, and stool secretory Ig A (sIgA) levels can also be elevated due to inflammation or CRC [11,23]. Studies indicate interplay between inflammation mediated by the immune system and the microbiota in the progression of CRC. For example, researchers found that germ-free mice did not develop colon tumors when treated with a mutagen while those with a bacterial infection resulting in increased inflammation developed more tumors after mutagen treatment than uninfected, normally colonized mice [18]. It is unknown whether groups at higher risk for CRC, such as AAs, have higher incidence of underlying intestinal inflammation than people from other racial/ethnic groups. 

Stool can provide an overall picture of the intestinal microbiota composition, bacterial metabolite production and the current inflammatory status of the colon [24,25]. Studies suggest that combining recommended CRC screening methodologies with detection of stool inflammatory markers, such as lactoferrin and sIgA, may increase the sensitivity and specificity of adenoma and CRC detection [11,26]. The relationship between comprehensive stool analysis (CSA) and colonoscopy findings may reveal significant potential for assessment of clinical risk and targets for dietary intervention.

Investigations into the intersection of behavior, environment, intestinal microbiota, bacterial metabolites and genetics will promote understanding of cancer progression and may lead to targeted approaches for eliminating racial/ethnic colorectal cancer disparities. The aim of this study was to explore how colonoscopy findings relate to dietary intake and stool SCFA levels and markers of inflammation in a diverse group of average risk adults scheduled for routine screening colonoscopies.

## 2. Materials and Methods 

### 2.1. Recruitment

Patients at normal risk for CRC were recruited after scheduling a CRC screening colonoscopy at the University of Kansas Health System Endoscopy Unit. Participants were recruited into two consecutive pilot studies occurring between 2012–2014 and 2014–2016. Protocols were reviewed and approved by the Institutional Review Board at the University of Kansas Medical Center (FWA# 00003411; project identification #13266 and #13370). Eligibility criteria included age 50–75, home address, access to a working telephone and willing to perform study requirements. Exclusion criteria included fecal occult blood test (FOBT) within one year, sigmoidoscopy or barium enema within five years, or colonoscopy within 10 years, acute medical illness, current gastrointestinal bleed, history of adenomatous polyps, CRC, first degree relative with CRC before age 60, inherited polyposis/non-polyposis syndrome, inflammatory bowel disease, cognitive impairment or inappropriate affect or behavior or another household member enrolled in the study.

Upon scheduling with endoscopy, potential participants were sent an opt-out letter introducing the study and signed by their gastroenterologist. Potential participants were asked to email or call study staff if they did not wish to be contacted. Scheduled patients who did not opt-out were called by study staff and evaluated for interest and eligibility. Interested and eligible patients met study staff at the academic medical center and were guided through written informed consent, completed a brief demographic and health history survey and were given the Diet History Questionnaire II (DHQ II) and a kit to collect stool to be sent for CSA. Participants were instructed to complete the DHQ II at home before their colonoscopy and to collect their stool according to kit instructions within the five days prior to their colonoscopy and before initiating colonoscopy preparation. Participants returned their DHQ IIs to study staff at their colonoscopy appointment and sent their stool samples directly to Doctor’s Data, Inc., in a postage paid envelope [14]. 

### 2.2. Comprehensive Stool Analysis

The Doctor’s Data CSA is used to detect a number of variables in stool including culturable species of bacteria and yeast, short chain fatty acids produced by bacteria in the gut and enzymatic and immunological markers of inflammation. For this study, we recorded SCFA information (the total level of detected SCFAs in mg/mL of stool, as well as the levels and proportions of the following individual SCFAs: acetate, propionate, butyrate and valerate) and three markers of inflammation (lactoferrin (µg/mL), lysozyme (ng/mL) and sIgA (mg/dL)). SCFAs were detected by gas chromatography and inflammatory markers were detected by enzyme-linked immunosorbent assay (ELISA). Inflammatory markers were categorized as abnormally high, normal, or abnormally low as per Doctor’s Data reference ranges.

### 2.3. Dietary Intake Analysis

At recruitment, participants were given a paper-based US National Cancer Institute DHQ II to report food and beverage intake and portion size over the past year. Total dietary fiber (grams/day), total fruit (cup equivalents/day; not including fruit juice) and total vegetable (cup equivalents/day) intake estimates were then quantified using analysis software (Diet*Calc version 1.5.0, National Cancer Institute, Bethesda, MD, USA). To more thoroughly evaluate dietary factors that are known to influence SCFA levels, an additional dietary variable, non-starchy vegetables (cup equivalents/day), was calculated by subtracting two Diet*Calc generated variables, ‘white potato’ and ‘other starchy vegetable,’ from the ‘total vegetable’ variable. 

### 2.4. Colonoscopy Findings

Participants underwent colonoscopy screening at the University of Kansas Health System Endoscopy Unit. Medical record data on completed colonoscopies and pathology findings were obtained from the medical center electronic health record. The presence or absence of adenomatous polyps was recorded for each participant. 

### 2.5. Statistical Evaluation

Two-tailed independent samples *t*-tests, or Mann-Whitney tests if necessary, were used to compare total SCFA, acetate, propionate, butyrate and valerate levels, colonoscopy completion and fiber, fruit and vegetable consumption in non-Hispanic African Americans versus other race/ethnicities. Participants were classified by their self-reported, combined race and ethnicity as non-Hispanic AA, non-Hispanic White, Hispanic and non-Hispanic Other. A dichotomous race/ethnicity variable was created to distinguish non-Hispanic AAs and all other race/ethnicities (Others). A 0.05 significance level was used. Pearson correlations among SCFA levels and diet variables were computed.

The relationship between race/ethnicity and total SCFA levels was investigated through a linear regression model with total SCFA levels as the dependent variable and race/ethnicity, diet, gender, age and smoking status as independent variables. A backward selection procedure was used to build linear regression models of total SCFA.

A frequency table of abnormal colonoscopies by race/ethnicity was built and a Fisher’s exact test was conducted at a 0.05 significance level to test the null hypothesis that non-Hispanic African-Americans and Others are equally likely to have abnormal colonoscopy findings. 

A logistic regression model of abnormal colonoscopies was fitted. The dichotomous dependent variable of this model was having or not having adenomatous polyps. The independent variables of the model included: race/ethnicity, total SCFAs, diet, gender, age and smoking status. Analogous logistic regression models were fitted, using butyrate levels and relative levels of butyrate and valerate in place of total SCFA levels. 

## 3. Results

### 3.1. Participants

Participant characteristics are listed in Table 1. AAs in our population were significantly younger and had significantly lower educational attainment than participants from other race/ethnicities (Table 1). There were not significant differences between AAs and other race/ethnicities in the distributions of body mass index (BMI), sex, smoking status and marital status. Not all participants completed all study activities. Forty-eight participants completed the demographic and health history survey, forty-four submitted stool for the CSA and forty-three completed the diet survey and colonoscopy screening.

### 3.2. Comparisons of Short Chain Fatty Acid Levels and Diets in African-Americans versus Others

**Figure 1:**
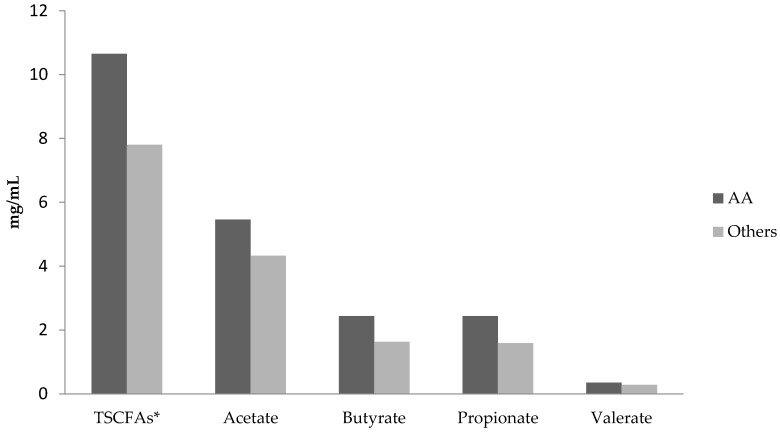
Levels of total short chain fatty acids (TSCFAs) and individual SCFAs for African Americans versus those of other race/ethnicities. All values in mg/mL of stool. * Statistically significant difference by linear regression, *p* = 0.029.

Mean total SCFA levels in AAs were higher than in Others (10.7 versus 7.8 mg/mL; Figure 1). A linear regression model of total SCFAs with only race/ethnicity as the independent variable showed total SCFA levels were significantly higher in AAs (*p* = 0.028). There were not significant differences in acetate, propionate, butyrate, or valerate levels between AAs and Others (Figure 1). AAs reported consuming significantly fewer non-starchy vegetables than others (*p* = 0.02; Table 2). However, this difference became non-significant after a Bonferroni correction for the four comparisons in Table 2. Moreover, there were not significant differences in fiber and fruit consumption between AAs and Others (Table 2). Other factors examined as independent variables in regression models were gender, age, current smoking and colonoscopy completion. None of these variables were significantly correlated with SCFA levels or dietary intake.

Total SCFA levels were not significantly correlated with fiber, fruit, vegetable, or non-starchy vegetable consumption (not shown).

### 3.3. Analysis of the Effects of Fruit and Vegetable Consumption on Total Short Chain Fatty Acids

In a linear regression model of total SCFA levels, vegetable consumption and the interaction between race/ethnicity and vegetable consumption were not significant (data not shown). Similarly, non-starchy vegetable consumption did not significantly affect total SCFA levels and did not interact significantly with race/ethnicity (not shown).

We explored the relationship of dietary vegetable consumption with total SCFA to see whether SCFA levels were always higher for AAs at any level of vegetable consumption. Using linear regression, we computed predicted values of total SCFA for particular percentiles of vegetable consumption stratifying by race/ethnicity. After adjusting for vegetable consumption, AAs tended to have higher SCFA values than non-AAs, although the interaction was not significant (not shown).

Total dietary fruit consumption did not have a significant effect on total SCFA levels (data not shown). We computed predicted values of total SCFA for particular percentiles of fruit consumption, stratifying by race/ethnicity. After adjusting for fruit consumption, AAs tended to have higher SCFA values than non-AAs, although the interaction was not significant (not shown).

### 3.4. Correlations between Inflammatory Markers, Short Chain Fatty Acid Levels, Diet Variables and Race/Ethnicity

We identified a significant positive correlation between stool lysozyme levels and acetate levels (r = 0.34, *p* = 0.028), however, this relationship is not significant after a Bonferroni correction for multiple comparisons. No significant relationships were found between stool lactoferrin or sIgA levels and stool SCFA levels.

There were not significant differences in lysozyme or lactoferrin levels between AAs and other race/ethnicities (two-tailed two-sample *t* test *p* ≥ 0.23; Mann-Whitney *p* ≥ 0.20). After adjusting for non-starchy vegetable consumption, AAs had a borderline significant likelihood of having low sIgA values (*p* = 0.059). However, race/ethnicity was not significantly associated with sIgA levels after adjusting for the other diet variables, presence of polyps or other potential confounders. 

Stool inflammatory marker levels were not significantly correlated with fiber consumption, fruit consumption or non-starchy vegetable consumption.

### 3.5. Comparison of Abnormal Colonoscopy Results in African-Americans versus Other Race/Ethnicities

In our study population, African-Americans and Others were equally likely to have abnormal colonoscopy results (two-sided Fisher’s exact, *p* = 0.99).

### 3.6. Analysis of the Relationship between Abnormal Colonoscopy Results and Race/Ethnicity, Short Chain Fatty Acids, Inflammatory Markers and Diet

To examine the likelihood of having abnormal colonoscopies, logistic regression models of abnormal colonoscopies were fitted using gender, race/ethnicity, smoking status, diet, age and stool inflammatory markers and SCFA levels as potential independent variables that were examined through a backward selection procedure. The interaction between race/ethnicity and SCFA levels was also examined. None of the investigated variables exhibited significant effects on the likelihood of having abnormal colonoscopies (not shown). Although not significant, we observed a trend of higher stool sIgA levels in participants who completed colonoscopy with adenomatous polyps compared to those without polyps (Table 3).

## 4. Discussion

Here, we present a snapshot of the colonic environment of participants at average risk for CRC at the time of a screening colonoscopy. Our study found that AAs had higher stool levels of SCFAs than others but were not significantly different in their likelihood of having abnormal colonoscopies. Relationships among race/ethnicity, diet, SCFAs, inflammatory markers and colonoscopy findings were unremarkable. While these findings do not help clarify our understanding of the interplay between factors related to CRC development in AAs and are not consistent with findings on the protective effects of SCFAs in vitro and in mouse models, they are consistent with findings among AAs with and without adenomatous polyps [15]. To further explore these relationships and ensure representativeness of data, larger sample size studies with longitudinal data are clearly needed.

SCFA are produced by colonic bacteria and subsequently absorbed by colonocytes. Research has shown that stool SCFA levels may not be fully representative of SCFA production and absorption rates [27,28]. Without data on plasma SCFA levels, it is unclear whether higher SCFA levels in stool from AAs in our study population are due to increased production of SCFA or decreased absorption. Further studies into SCFA absorption and the role of gut-barrier function may shed light on increased risk of CRC in AAs. BMI may be another possible explanation for increased stool SCFA levels for AAs in our study population. Studies suggest that overweight and obese individuals produce more colonic SCFAs than lean individuals, independent of diet and SCFA absorption [29]. BMI may account for the increased stool SCFA levels of AAs in our population. Although not statistically significant, AAs in our study population had higher mean BMIs than other race/ethnicities (Table 1). We also evaluated caloric intake in our analyses and found no statistically significant difference in intake among AAs and other race/ethnicities (data not shown).

The question of biology versus environment as a factor in CRC incidence and mortality differentials across racial/ethnic groups does not have a straightforward answer. CRC tumorigenesis is likely multifactorial. Native Africans have lower rates of colon cancer than Caucasian Africans, while AAs have the highest incidence among racial/ethnic groups in the United States [30,31,32]. High CRC incidence in AAs has been attributed to demographic factors such as socio-economic status and access to healthcare, yet Hispanics have similar demographic factors while experiencing much lower CRC incidence than AAs [33,34]. Racial and ethnic differences exist in dietary intake among cancer survivors: non-Hispanic AAs have the lowest intake of vegetables, whereas Hispanics have the highest mean intake of vegetables [35]. Dietary fiber from fruit, vegetables and whole grains may act in various ways to reduce CRC progression, and the protective effects are largely mediated by production of metabolites by the gut microbiota [36]. Fiber serves as a substrate for the production of SCFAs by intestinal bacteria. One SCFA, butyrate, inhibits colonic cell proliferation and increases tumor suppressor gene activation [37]. Some research suggests an increased sensitivity of cancerous colonic cells to the anti-proliferative effect of butyrate [38]. Others have argued that the absence of CRC promoting dietary factors (animal protein and fat) rather than the abundance of fiber in the diet have a greater impact on CRC risk [32]. For example, high dietary fat leads to a buildup of bile acid deoxycholic acid (DCA) in the lumen of the colon, which has been shown to reduce tumor suppressor activation and apoptosis pathways in colonic cells [37]. The comprehensive stool assessment used in this study did not measure bile acids, therefore buildup of DCA was not investigated on our study population. 

In addition to providing a fiber substrate for colonic bacteria, dietary intake of fruits and vegetables has been shown epidemiologically and clinically to play a protective role against CRC due to the chemopreventive effects of phytochemicals [39,40]. Low intake of vegetables, as seen in the AAs in our study population, reduces the number of phytochemicals available to rapidly proliferating cells in colonic crypts. Phytochemicals from diet serve as the substrate for production of antioxidants, phytoestrogens and anti-inflammatory agents that have been implicated in providing health benefits and reduction of CRC risk [39,40]. 

Very little is known about racial/ethnic differences in stool inflammatory markers, such as lysozyme, lactoferrin and secretory IgA. Salivary and serum sIgA have been shown to differ between racial/ethnic groups in a few conflicting studies. One study found lower salivary sIgA in older AA men taking hypertensive medications [41]. However, mean total salivary sIgA levels were found to be significantly higher in AA women than Caucasian women [42]. Elevated serum IgA has been detected in AAs compared to others [43].

Our findings are preliminary and not generalizable due to our small sample size; however, our results contribute to the growing evidence showing the complex interplay between protective and harmful factors in the colon. Additional exploration of the microbiota composition detected in stool and the influence of diet on microbial metabolite production will be critical to inform personalized therapeutic and preventive recommendations, particularly in the context of human genetics influencing susceptibility to CRC. Contradictory results from a variety of studies point to a need for identification of additional signaling factors in the polyp and tumor developmental pathway and more in-depth exploration of the direct and indirect role of the microbiota in the prevention and promotion of CRC. Future studies evaluating the abundance of informative factors available from stool samples, the characterization of the microbiome of stool and mucosal adherent bacteria, as well as the role of genetic and environmental factors influencing signaling and proliferation of normal and abnormal tissue are needed. We hope the findings we present here will contribute to the planning and conduct of such studies.

## Figures and Tables

**Table 1:** Participant characteristics.

Characteristic	AA (*n* = 17)	Other (*n* = 31)	Overall
Age, mean (range) ^a^	53.5 (50–67)	58.7 (49–71)	56.9 (49–71)
BMI, mean ± SD ^b^	33.5 ± 8.6	30.1 ± 7.0	31.3 ± 7.7
Race/ethnicity, *n* (%)			
Non-Hispanic AA	17 (100)	0	17 (35)
Non-Hispanic White	0	24 (77)	24 (50)
Hispanic	0	7 (23)	7 (15)
Sex, *n* (%)			
Male	9 (53)	14 (45)	23 (48)
Female	8 (47)	17 (55)	25 (52)
Smoking status, *n* (%)			
Currently smokes	6 (35)	6 (19)	12 (25)
Used to smoke	2 (12)	7 (23)	9 (19)
Never smoked	9 (53)	18 (58)	27 (56)
Marital status, *n* (%) ^c^			
Married/living with partner	7 (44)	16 (52)	23 (49)
Divorced or separated	5 (31)	8 (26)	13 (28)
Widowed or never married	4 (25)	7 (22)	11 (23)
Educational attainment, *n* (%) ^c,d^			
≤High school	8 (50)	5 (16)	13 (28)
≥College	8 (50)	26 (84)	34 (72)

^a^ African Americans (AA) participants were significantly younger than participants from other race/ethnicities (*p* = 0.004 by 2-tailed independent samples *t*-test and 0.002 by Mann-Whitney test). ^b^ Two subjects (one AA and one non-AA) did not provide body mass index (BMI) data. ^c^ One AA participant did not provide information about education or marital status. ^d^ AA participants had significantly lower educational attainment than participants from other race/ethnicities (*p* = 0.02 by Fisher’s Exact test).

**Table 2:** Comparison of dietary intakes in African Americans versus other race/ethnicities.

Diet Variable ^a^	AA (*n* = 14) ^b^	Other (*n* = 29) ^b^
	mean (95% CI)	mean (95% CI)
Fiber, g/d	25.26 (11.39, 39.13)	23.08 (19.50, 26.66)
Fruit, c/d	2.21 (0.88, 3.55)	1.52 (1.03, 2.00)
Total vegetable, c/d	1.65 (0.96, 2.34)	2.39 (1.75, 3.03)
Non-starchy vegetable, c/d *	1.11 (0.62, 1.61)	2.00 (1.42, 2.58)

^a^ g/d = grams/day; c/d = cup equivalents/day. ^b^ Three AA participants and 2 participants of other race/ethnicity had missing values in the diet variables and were not included in these computations. * Statistically significant by two sided-*t*-test (*p* = 0.02) and Mann-Whitney test (*p* = 0.03).

**Table 3:** Comparison of participants with adenomatous polyps to those without, among participants who completed colonoscopy ^a,b^.

	Absence of Adenomatous Polyps	Presence of Adenomatous Polyps	Overall
**Race/ethnicity, *n* (%)**	*n* = 29	*n* = 14	*n* = 43
Non-Hispanic AA	9 (31)	4 (29)	13 (30)
Non-Hispanic White	15 (52)	8 (57)	23 (53)
Hispanic	5 (17)	2 (14)	7 (16)
**SCFA, mean (95% CI) ^c^**	*n* = 26	*n* = 13	*n* = 39
Total, mg/mL	8.90 (7.11, 10.69)	8.58 (6.74, 10.42)	8.79 (7.50, 10.09)
Acetate, mg/mL	4.75 (3.97, 5.53)	4.40 (3.57, 5.23)	4.63 (4.06, 5.20)
Butyrate, mg/mL	2.04 (1.41, 2.67)	1.87 (1.28, 2.46)	1.98 (1.54, 2.43)
Propionate, mg/mL	1.82 (1.33, 2.32)	2.03 (1.37, 2.69)	1.89 (1.51, 2.27)
Valerate, mg/mL	0.29 (0.25, 0.35)	0.30 (0.21, 0.38)	0.30 (0.25, 0.34)
**Dietary intake, mean (95% CI) ^d,e^**	*n* = 27	*n* = 13	*n* = 40
Total fiber g/d	27.1 (20.4, 33.8)	20.9 (14.9, 26.9)	25.1 (20.2, 30.0)
Total fruit c/d	2.0 (1.3, 2.8)	1.4 (0.6, 2.2)	1.8 (1.3, 2.4)
Total vegetable c/d	2.3 (1.6, 2.9)	2.3 (1.3, 3.2)	2.3 (1.8, 2.8)
Non-starchy vegetable c/d	1.9 (1.3, 2.5)	1.7 (1.0, 2.4)	1.8 (1.4, 2.3)
**Inflammatory markers, mean (95% CI) ^f^**	*n* = 26	*n* = 13	*n* = 39
Lysozyme, ng/mL ^g^	249.2 (164.1, 334.2)	255.1 (149.3, 360.8)	251.2 (187.3, 315.1)
Lactoferrin, µg/mL	5.1 (1.7, 8.5)	2.4 (0.13, 4.7)	4.2 (1.9, 6.5)
sIgA, mg/dL	160.3 (103.0, 217.7)	307.2 (98.3, 516.2)	209.3 (132.6, 286.0)

^a^ Four non-Hispanic AA participants and one non-Hispanic white participant did not complete colonoscopy; ^b^ At a 0.05 significance level, the two populations were not significantly different in any of the reported variables; ^c^ Four subjects who completed colonoscopy did not provide SCFA data (3 of them did not exhibit adenomatous polyps, 1 exhibited polyps); ^d^ g/d = grams/day; c/d = cup equivalents/day; ^e^ Three subjects who completed colonoscopy did not provide dietary intake data (2 of them did not exhibit adenomatous polyps, 1 exhibited polyps); ^f^ Four subjects who completed colonoscopy did not provide inflammatory marker data (3 of them did not exhibit adenomatous polyps, 1 exhibited polyps); ^g^ After excluding an outlier, the mean lysozyme level for subjects without polyps was 219.2 (95% CI, (158.3, 280.1)). sIgA: secretory IgA.
